# Follicular Bronchiolitis: Two Cases with Varying Clinical and Radiological Presentation

**DOI:** 10.1155/2020/4564587

**Published:** 2020-01-25

**Authors:** Delyse Garg, Mohit Mody, Chaitanya Pal, Pratik Patel, Christina Migliore, Christine Minerowicz, Nikhil Madan

**Affiliations:** ^1^Division of Pulmonary and Critical Care, Newark Beth Israel Medical Center, Newark, NJ 07112, USA; ^2^Department of Medicine, Newark Beth Israel Medical Center, Newark, NJ 07112, USA; ^3^Department of Pathology, Rutgers Robert Wood Johnson Medical School, New Brunswick NJ 08901, USA

## Abstract

Follicular bronchiolitis (FB) is a rare bronchiolar disorder associated with hyperplasia of the bronchial-associated lymphoid tissue (BALT). It is characterized by the development of lymphoid follicles with germinal centers in the walls of small airways. It falls under the category of lymphoproliferative pulmonary diseases (LPDs) and commonly occurs in relation to connective tissue disease, immunodeficiency, infections, interstitial lung disease (ILD), and inflammatory airway diseases. Computerized tomography (CT) findings include centrilobular nodules with patchy ground glass infiltrate, tree-in-bud findings, and air trapping. It can very rarely present as diffuse cystic lung disease. We present two cases of FB. The first case is associated with Human Immunodeficiency Virus (HIV) infection and asthma with diffuse cystic changes on the CT. The second case is associated with reactive airway disease and gastroesophageal reflux disease (GERD) with the classic centrilobular nodules and ground glass opacities on the CT.

## 1. Introduction

One of the first reports of excessive lymphoid follicular formation in the diseased bronchioles was reported in 1952 by Whitewall when describing bronchiectasis [1]. Follicular bronchiolitis and its relation to bronchial-associated lymphoid tissue (BALT) was first described by Bienenstock et al. in 1973. It is currently classified as a benign lymphoproliferative pulmonary disease (LPD) [2]. It is characterized by the development of lymphoid follicles with germinal centers in walls of the small airways. It is thought to be caused by antigenic stimulation and hyperplasia of BALT throughout the airway. This may be the only characteristic finding, but in some cases, it can cause bronchiectasis and, rarely, present as a cystic lung disease. It can be primary/idiopathic or secondary and is associated with connective tissue disease, immunodeficiency states, and infections [3].

## 2. Case 1

The first patient was a 24-year-old African American female with past medical history of congenital Human Immunodeficiency Virus (HIV) infection, compliant with antiretroviral therapy (ART) with a CD4 count of 275, recently diagnosed endometriosis, and childhood asthma. She was admitted to the hospital for an elective dilatation and curettage procedure. She was seen postprocedure by pulmonary medicine for acute onset of shortness of breath. She was noted to be in mild respiratory distress with oxygen saturation of 96% on 4 liters of oxygen via nasal cannula but, otherwise, had a normal exam. She improved after receiving nebulized albuterol. Upon further questioning, the patient revealed that she was diagnosed with asthma as a child. She was treated with various inhaled and nebulized medications throughout her childhood. During early adolescence, she required home oxygen for about 1-2 years. She had multiple hospitalizations for presumed asthma exacerbation during her childhood and teenage years. She did not recall having a pulmonary function test or any imaging of the chest. She had never been placed on noninvasive or invasive mechanical ventilation. Over the last 5-6 years, she had no hospitalizations for shortness of breath. She did not use oxygen at home. She had a desk job as a receptionist in a doctor's office so was able to perform her duties without experiencing any respiratory distress. However, she did get short of breath after walking 3 blocks and after climbing 1 flight of stairs. She used an albuterol inhaler which she said brought only mild relief of her symptoms. She had never smoked cigarettes or any other illicit drugs. However, she was exposed to second-hand smoke as her grandmother and several of her friends smoked in her presence.

**Figure 1:**
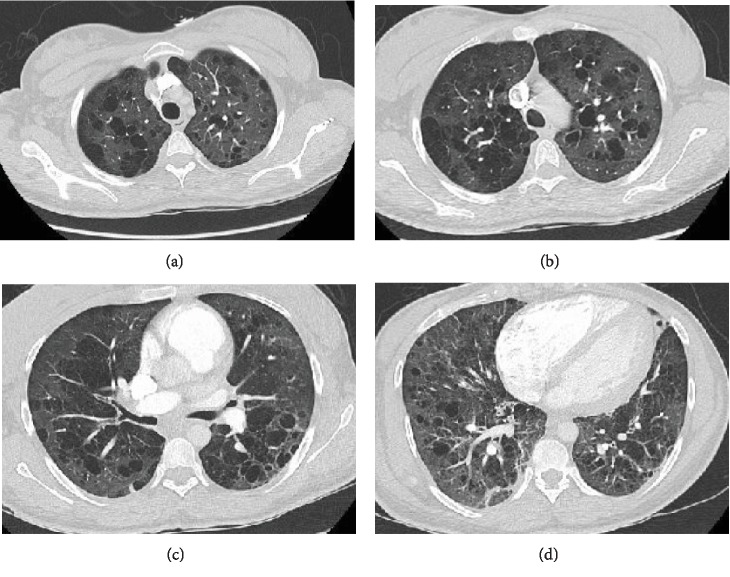
(a–d) CT chest with severe cystic disease involving all lobes with peribronchial thickening and cylindrical bronchiectasis at the bases.

Her hemoglobin on presentation was 8.9 g/dL, stable as compared to previous levels. She had a normal white blood cell, platelet count, and renal function. A liver function test was normal except for a low albumin level of 3.0 g/dL. Her lactic acid level was normal. An arterial blood gas showed pH of 7.38, pCO_2_ of 37 mmHg, and pO_2_ of 70 mmHg on room air. Her pulmonary function test showed FEV1 of 0.9 liters (33% of predicted), FVC of 1.9 liters (61% of predicted), and diffusion capacity of 8% predicted. A CT (Figure 1) showed severe bilateral cystic changes involving peribronchial thickening and cylindrical bronchiectasis at the bases. There was no evidence of pulmonary emboli. There were no masses or enlarged lymph nodes.

The patient's evaluation for cystic lung disease included a negative folliculin gene test, ruling out Birt-Hogg-Dubé syndrome, and a normal serum vascular endothelial growth factor-D (VEGF-D) level arguing against lymphangioleiomyomatosis. Her autoimmune testing was negative as well.

**Figure 2:**
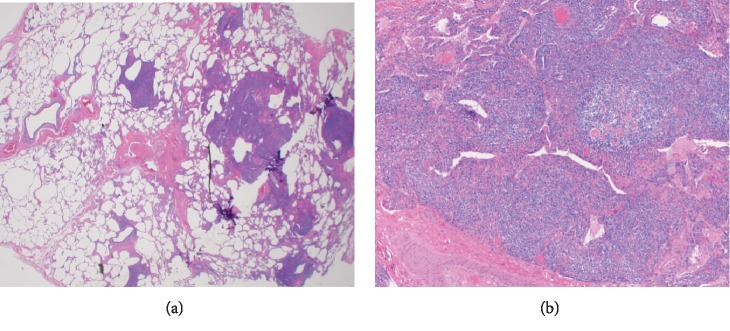
(a) Low-power image of the lung showing marked airway-centric inflammation (left side of image). Centrilobular emphysema is also present (right side of image) (H&E, 1.25x). (b) High-power image of bronchiole. There is a nodular lymphocytic infiltrate of small airways with germinal center formation (H&E, 20x).

She then underwent right-sided video-assisted thoracoscopic surgery with right upper, middle, and lower lobe wedge resections and biopsy. Tissue samples were negative for fungal growth and tuberculosis. Pathologic examination of the lung revealed marked follicular bronchiolitis and centrilobular and subpleural cystic changes. There was no evidence of a malignant lymphoproliferative disorder (Figures 2(a) and 2(b)).

She was started on 60 mg of prednisone daily. However, after two months of treatment, she had no improvement in respiratory symptoms and began to develop complications from steroid use such as weight gain, myalgias, and hyperglycemia. The prednisone was tapered slowly, and a trial of azithromycin was started; however, she failed to respond to that as well. She was still able to perform her daily activities without the need for supplemental oxygen. She was referred to the lung transplantation clinic for further evaluation.

## 3. Case 2

**Figure 3:**
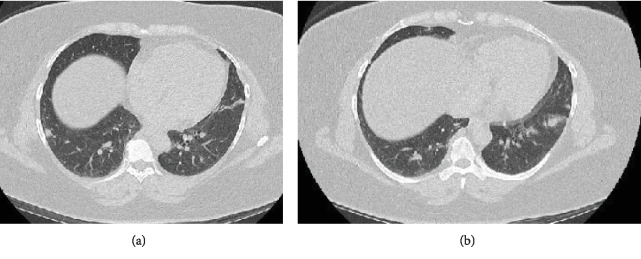
(a, b) Multiple pulmonary nodules, with the largest seen in the lower lobes.

**Figure 4:**
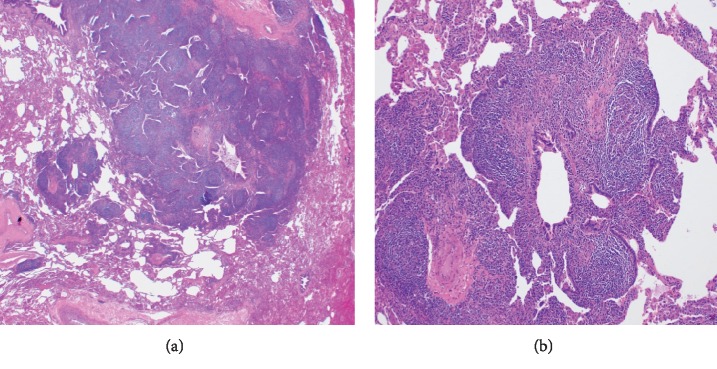
(a) Low-power image of the lung showing numerous lymphoid follicles with germinal centers surrounding the small airways. The interstitium away from the airways is relatively spared of inflammation, and cyst formation is not prominent. (b) High-power image of the bronchiole is similar to patient 1.

The second case was of a 45-year-old morbidly obese Hispanic female with a past medical history of reactive airway disease and gastroesophageal reflux disease (GERD) who presented to the outpatient pulmonary clinic with a 5-month history of worsening shortness of breath and nonproductive cough. Her symptoms were insidious in onset and gradually progressed to dyspnea at rest. She denied recent travel, sick contacts, active or passive smoking, exposure to animals, or other inhaled pollutants. Vital signs were remarkable for a low-grade fever (100.2°F), tachypnea with a respiratory rate of 20 breaths/minute, and a peripheral oxygen saturation of 92% on room air. Physical examination revealed coarse crackles throughout both lung fields and digital clubbing. Laboratory analysis was unremarkable and did not suggest an infectious or rheumatologic etiology for the dyspnea. A chest radiograph showed hyperinflated lungs with diffuse interstitial infiltrates. CT scan of the chest (Figure 3) revealed bilateral noncalcified nodular opacities, the largest measuring approximately 1 cm in size. Pulmonary function testing showed normal spirometry and lung volumes with a positive bronchodilator response and diffusion capacity of 80% of predicted. A 6-minute walk test revealed desaturation to 86%, which improved to 92% with administration of 2 L oxygen via nasal cannula. A transesophageal echocardiogram (TEE) visualized a 1.8 cm atrial septal defect (ASD) with intermittent shunting and a mildly dilated right ventricle. Right heart catheterization was resulted as borderline pulmonary hypertension with a mean pulmonary artery pressure of 25 mmHg, pulmonary capillary wedge pressure of 20 mmHg, and cardiac output and cardiac index of 7.3 L/min 3.8 L/min/m^2^, respectively. As her symptoms continued to worsen, she underwent a follow-up CT scan at 2 months and then at 4 months after presentation, with persistent bilateral nodular opacities, grossly unchanged in size. She initially underwent a bronchoscopy with electromagnetic navigation and transbronchial lung biopsy of the lower lobe nodules which was inconclusive upon pathological analysis. Video-assisted thoracoscopic surgery (VATS) with wedge biopsy of the left upper lobe was performed. Pathologic examination showed exuberant reactive follicular lymphoid hyperplasia with germinal centers predominantly associated with the small airways. There was no morphologic or immunophenotypic evidence of hematologic malignancy (Figure 4). Flow cytometry studies were also negative. Lymphocytic interstitial pneumonia (LIP) was not favored as there were only few, small foci of interstitial chronic inflammation. There were no typical findings of asthma-related changes such as eosinophils, thickened basement membranes, or goblet cell hypertrophy. Pulmonary nodular lymphoid hyperplasia was not favored due to the number of lymphoid nodules in the specimen and in the CT scan.

## 4. Discussion

Follicular bronchiolitis (FB) is classified as a reactive pulmonary lymphoid disorder and is part of a group called the lymphoproliferative pulmonary diseases (LPDs). Subepithelial accumulation of lymphoid follicles in the affected bronchioles causes distortion of the architecture of the bronchial tree and can lead to obstructive airway disease [3].

Follicular bronchiolitis is classified based on presentation and the presence or absence of underlying systemic disease. Secondary FB is seen in patients with immunodeficiency syndromes such as Common Variable Immune Deficiency (CVID) and Acquired Immunodeficiency Syndrome (AIDS), in association with several autoimmune diseases, along with infections such as active hepatitis, *Legionella*, or Pneumocystis pneumonia, and even in conjunction with other ILDs [3]. Idiopathic FB is rare and occurs sporadically, usually in middle-aged and elderly individuals, who present with progressively worsening dyspnea and sometimes dry cough. The secondary form can occur at any age. The manifestations of the underlying disorder are usually evident before pulmonary symptoms [4].

Chest radiography is usually normal but can show evidence of reticulonodular infiltrates or hyperinflated lungs due to air trapping [5]. Centrilobular nodules ranging from 1 to 3 mm in diameter are the most commonly encountered abnormality on a high-resolution CT scan. It can give the appearance of a tree-in-bud. However, there can also be evidence of bilateral patchy ground glass opacities as well as significant air trapping [6, 7]. Very rarely, FB can present as cystic lung disease and can be confused with LIP. The cyst formation is thought to be secondary to ischemia due to vascular obstruction, post obstructive bronchiolar ectasia, or bronchiolar compression by lymphoid tissues. Pulmonary function testing in patients with FB is often nonspecific and can reveal a normal, restrictive, obstructive, or mixed pattern [3].

The diagnosis of FB is histopathological and requires the demonstration of multiple lymphoid follicles with germinal centers in the bronchiolar walls, as well as narrowing of the bronchiolar lumen. It is differentiated from LIP by the relative sparing of the alveolar septa in FB [8, 9]. Other features include foci of organizing pneumonia and sometimes an intraluminal neutrophilic infiltrate [10].

Treatment strategies include management of the underlying or associated disease, such as immunosuppressive therapy for autoimmune disease. Initiating antiretroviral therapy in a patient with HIV-associated FB has shown improvement in respiratory symptoms [11]. In patients with CVID, intravenous immunoglobulin replacement therapy has been used, as well as immunotherapy with rituximab and azathioprine to good result [12, 13]. Unfortunately, treatment options for idiopathic FB are limited to the use of corticosteroids and macrolide antibiotics with varying responses. They have demonstrated good results when started early on in the course of the disease; however, there is no good therapy for advanced lung disease. Overall prognosis is good in primary FB in the middle aged and elderly. Patients with secondary FB tend to do worse in general. Given the rarity of the disease, there are no treatment guidelines in place. The progression to advanced lung disease in FB associated with HIV despite adequate immunosuppression as noted in the first case is of particular interest. To our knowledge, this has not been reported.

The role of lung transplant for advanced lung disease from FB is not well studied. To our knowledge, there are no case reports of lung transplant for advanced lung disease from FB. Evaluation for lung transplant should be considered in FB with advanced lung disease. FB has been recognized as a rare cause of bronchiolitis obliterans syndrome after lung transplantation [14].

## 5. Conclusion

FB is a rare bronchiolar disorder with varying clinical and radiological presentations. These cases highlight the varying presentations of this disease entity. Clinicians should consider this as differential, especially when evaluating patients with infectious, inflammatory, and immunodeficiency states and autoimmune disease processes. Although extremely rare, it is a known cause of cystic lung disease and should be considered. Overall prognosis for FB is good. Currently, there are no treatment guidelines for idiopathic FB. Treatment of the underlying condition remains the mainstay in secondary FB.
